# Association Between Dietary Inflammatory Index and NAFLD: A Cross-Sectional Study of the National Health and Nutrition Examination Survey

**DOI:** 10.1155/mi/4954551

**Published:** 2025-07-01

**Authors:** Yuan He, Yuhang Yang, Pengfei Cheng, Wei Zhang, Jinghan Jia, Dawei Ye, Jinxi Wang

**Affiliations:** ^1^Department of Oncology, Tongji Hospital, Tongji Medical College, Huazhong University of Science and Technology, Wuhan 430030, China; ^2^Hepatobiliary Surgery, Baogang Hospital of InnerMongolia, Baotou 014000, China; ^3^Department of General Surgery, Third Hospital of Shanxi Medical University, Shanxi Bethune Hospital, Shanxi Academy of Medical Sciences Tongji Shanxi Hospital, Taiyuan 030032, China; ^4^Department of General Surgery, Shanxi Bethune Hospital, Third Hospital of Shanxi Medical University, Taiyuan 030032, China

**Keywords:** diet, dietary inflammation index, nonalcoholic fatty liver disease

## Abstract

**Background and Aim:** The aim of this study was to determine if there is an association between the dietary inflammatory index (DII) and nonalcoholic fatty liver disease (NAFLD).

**Methods:** Study data were obtained from the National Health and Nutrition Examination Survey (NHANES) 2017–2018. Multiple logistic regression models were used to assess the association between DII and NAFLD. A restricted cubic spline (RCS) was used to investigate the non-linear association between DII and NAFLD. A total of 8708 people were included, with no age limit.

**Results:** In fully adjusted multiple regression models, DII < 0 was associated with fewer incident NAFLD events compared with DII ≥ 0. In the RCS model, there was a positive nonlinear relationship between DII and NAFLD. In addition, the main positive association between DII and NAFLD was found in participants aged ≥60 years and who were white females.

**Conclusions:** A proinflammatory diet is associated with the development of NAFLD, and we recommend improving diet to reduce the risk of developing liver disease, especially NAFLD.

## 1. Introduction

Nonalcoholic fatty liver disease (NAFLD), a common chronic liver disease, a global prevalence of NAFLD was estimated to be 30.2% (95% CI: 28.7%–31.7%) [1]. The main cause of NAFLD is the development of metabolic syndrome in the body, which is associated with insulin resistance, obesity, and hyperlipidemia in the body [2]. Obesity and insulin resistance often lead to chronic inflammation; therefore, patients with NAFLD generally have chronic inflammation accompanying them. NAFLD is a part of the progression of liver disease that begins with steatosis in the liver and progresses to nonalcoholic steatohepatitis (NASH), which, without intervention, will proceed to the next step of advanced fibrosis, which may eventually lead to cirrhosis or even hepatocellular carcinoma. Early identification and intervention are particularly important given the adverse consequences of all of the above [3, 4].

Increased oxidative stress and inflammation induced by fatty deposits are considered to be the main drivers of hepatic and extrahepatic injury [5, 6]. On this basis, NAFLD is gradually undergoing development. There is no definitive drug that can successfully treat NAFLD, so new ways of treating NAFLD are constantly being explored in addition to drug treatment modalities. Numerous studies have reported that ingredients in many foods can reduce the risk of developing inflammation-related diseases [7–11]. There is a growing awareness of the importance of adopting good lifestyle habits to prevent or improve the development of diseases, and that liver diseases caused by fatty deposits can be prevented by losing weight or improving the quality of the diet. Diet and nutrition are increasingly recognized as major modifiable determinants of liver health and metabolic homeostasis [12]. Diet is a modifiable environmental factor that plays an important role in regulating inflammation and immune function, among other things. Previous studies have demonstrated that foods with anti-inflammatory properties, such as tea, vegetables, and nuts, can reduce the risk of developing rheumatoid arthritis [13, 14]. In addition, a summary of recent research on inflammatory diets and chronic diseases, such as diabetes and cardiovascular disease, found that diets with inflammatory features were strongly associated with the onset and severity of chronic diseases, and that some of these associations were gender-related [15]. In the face of a rich diet, rational choices play a vital role in promoting good health. By validating in vivo and in vitro models, Gao et al. [16] found that dietary intervention could enhance hepatic lipoautophagy by inhibiting the Akt/mTOR/ULK1 pathway and demonstrated for the first time that lipid accumulation increases hepatic senescence, and that hepatic senescence implies a decline in function, on the basis of which NAFLD is further progressed. Diet has a role in regulating inflammation, and making the right dietary choices can reduce inflammation in the body to a certain extent and even provide anti-inflammatory effects [17].

Dietary Inflammatory Index (DII) is a scoring mechanism derived from the literature to assess the inflammatory potential of diets and to link diet to inflammation [18]. The DII has been widely studied as a tool for measuring the inflammatory potential of diets [19–22]. The advantage of the DII over other diet quality scoring tools is that it has been validated against circulating concentrations of C-reactive protein (CRP) and other systemic markers of inflammation [18, 23].

The present study utilized data from the National Health and Nutrition Examination Survey (NHANES) database to assess the inflammatory potential of participants' diets through DII in order to investigate the effect of DII on NAFLD, and the effects of different factors on NAFLD, and to provide a theoretical basis for the prevention and treatment of NAFLD in life.

## 2. Methods

### 2.1. Study Population

**Figure 1:**
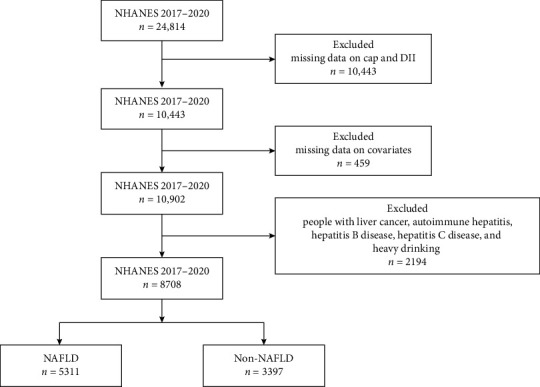
Flow chart of crowd selection. NHANES National Health and Nutrition Examination Survey. NAFLD, nonalcoholic fatty liver disease.

The NHANES database in the United States is responsible for compiling life and health statistics data [24]. NHANES collects data primarily through demographic, dietary, physical measurements, laboratory tests, and questionnaires. Due to the continuous updating of the NHANES database's inspection items, the outcome variables collected in this study have been collected since 2017. Therefore, the data collection and sample size determination have also been carried out since 2017. The final year we selected for inclusion in the population was 2017–2018. We excluded those with missing controlled attenuation parameter (CAP) score and DII (*n* = 10443), covariate (*n* = 459) information. Patients with other causes of chronic liver disease has also been excluded (*n* = 2194), including liver cancer, autoimmune hepatitis, hepatitis B disease, hepatitis C disease and heavy drinking (≥3 drinks/day for females, ≥4 drinks/day for males, or binge drinking ≥5 days/month) [25]. Finally, a total of 8708 participants were included in this study (Figure 1).

### 2.2. Outcome Variable

NAFLD refers to a clinical pathological syndrome characterized by excessive deposition of intracellular fat in liver cells, excluding alcohol and other clear liver-damaging factors. It is closely related to insulin resistance and genetic susceptibility, resulting in acquired metabolic stress-induced liver injury. Including simple fatty liver (SFL), NASH, and related cirrhosis. In this study, well-trained researchers performed liver ultrasound transient elastography on participants, which provided objective measurements of the manifestations of chronic liver disease and cirrhosis. When liver steatosis occurs, the device can record ultrasound attenuation related to steatosis and use the CAP as an indicator of liver fat [26]. We define CAP > 246 dB/m as NAFLD [27].

### 2.3. Explanatory Variables

Dietary information for the DII was obtained from participants' dietary recalls collected from the NHANES database for 2 days. The dietary data recorded 2 days of dietary memories and were collected by professionals. The first day's dietary data is collected at the mobile examination center (MEC), and after 3–10 days, professionals will collect the second day's dietary data from participants via phone. The detailed dietary intake is not suitable to be displayed as a table due to the large amount of data. Specific information can be found on the NHANES official website for display. For the calculation method of DII for the included population, refer to the study by Shivappa et al. [18]. Further numerical evaluation will be conducted based on whether the impact of each dietary parameter on six inflammatory markers (IL-1b, IL-4, IL-6, IL-10, TNF-a, and CRP) increases, decreases, or has no effect. For DII > 0, larger scores indicate that the dietary pattern is more prone to inflammation, and for DII < 0, smaller scores indicate that the dietary pattern has an anti-inflammatory effect [18].

### 2.4. Covariates

The covariates selected in this study include age, gender, race, marital status, education level, poverty ratio, smoking, diabetes, hypertension, body mass index, direct HDL-cholesterol, and triglyceride.

The covariates included in this study were categorized according to the classification criteria provided by the NHANES database. Marital status includes: married, never married, and widowed/divorced/separated.

The level of education includes: less than 9th grade, 9–11th grade, high school graduate/GED or equivalent, some college, and college graduate or above.

The poverty rate is a ratio of family income to the poverty threshold.

Categories of smoking: (1) never: smoked less than 100 times in one's lifetime, (2) former: smoked more than 100 times in one's lifetime and smokes not at all now, and (3) now: smoked more than 100 times in one's life, and currently smokes.

Diabetes can be diagnosed by any of the following: (1) being informed of diabetes mellitus by a doctor, (2) glycated hemoglobin ≥ 6.5%, (3) fasting glucose ≥ 7.0 mmol/L, (4) random blood glucose ≥ 11.1 mmol/L, (5) oral glucose tolerance test (OGTT) ≥ 11.1 mmol/L, and (6) taking antidiabetic drugs.

The specific values of body mass index, direct high-density lipoprotein cholesterol, and triglycerides can be directly obtained from the NHANES database [24].

The NCHS Institutional Review Committee approved the NHANES study, and all participants provided written informed consent forms.

### 2.5. Statistical Analysis

We conducted weighted analysis on all data using appropriate sampling weights (1/2 × WTMEC2YR from 2017 to 2020) to illustrate the complex survey design adopted by NHANES.

Categorical variables were expressed using (group ratios [standard errors]) and *X*2 test for comparisons between groups, and continuous variables were expressed using (means [standard errors]) and a *t*-test for comparisons between groups.

The relationship between DII and NAFLD was first examined using univariate regression analyses. Model 2 was adjusted for age, sex, and race, and Model 3 continued from Model 2 by adjusting for marital status, education level, poverty ratio, smoking, diabetes, hypertension, body mass index, direct HDL-cholesterol, and triglyceride.

In addition, subgroups and interactions were analyzed according to age group, sex, race, diabetes, and hypertension in logistic regression models that adjusted for all covariates. A restricted cubic spline (RCS) with four nodes was used in each logistic regression model to test the nonlinear relationship between DII and NAFLD.

All analyses were performed using R version 4.2.2. *p*  < 0.05 was considered statistically significant.

## 3. Results

### 3.1. Participant Characteristics

A total of 8708 people were enrolled in the study, including 5311 NAFLD patients and 3397 non-NAFLD patients. Figure 1 illustrates the process of population selection.  Table 1 demonstrates the demographic and clinical characteristics. The included population was 47.45% male, with the highest percentage of whites (36.15%) and the lowest percentage of Mexicans (11.28%) among the different races. About 22.24% are diabetics. The mean BMI for NAFLD patients was 32.45 compared to 25.83 for non-NAFLD patients.

### 3.2. Comparison Between Groups With and Without NAFLD

In Table 1, there are statistically significant differences (*p*  < 0.0001) in age, gender, race, marital, education, BMI, diabetes, hypertension, smoking, HDL-cholesterol, and triglyceride between patients with and without NAFLD.

### 3.3. DII is Independently Associated With NAFLD

Multivariate logistic regression analyses of DII and NAFLD are shown in Table 2. In Model 1, the risk of NAFLD for participants with DII < 0 was 0.84 times higher than for those with DII > 0. The same trend was observed in Model 3, which adjusted for age, gender, race, marital, education, smoking, diabetes, Hypertension, HDL-cholesterol, and triglyceride, with the risk increasing to 0.85 times.

**Figure 2:**
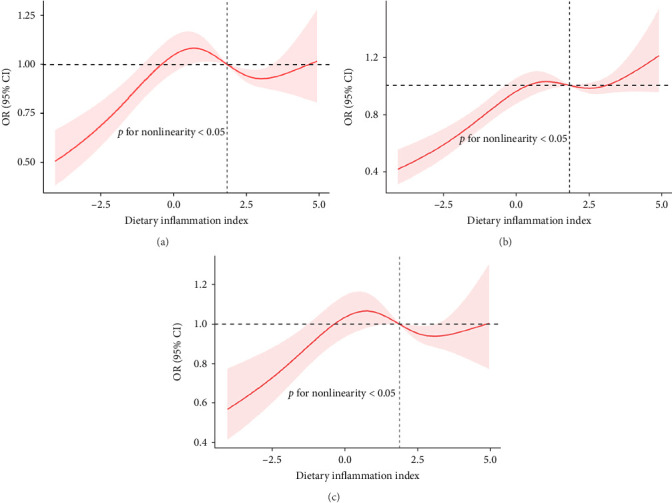
The relationship between DII and the risk of NAFLD. The nonlinear correlation between DII and NAFLD was studied using penalty cubic splines fitted in the logistic regression risk model. OR, odd ratio; CI, confidence interval: (a) Model Ⅰ, (b) Model Ⅱ, and (c) Model Ⅲ.

The RCS showed a nonlinear relationship between DII and NAFLD, with events of NAFLD increasing with DII (Figure 2).

### 3.4. Subgroup Analysis

**Figure 3:**
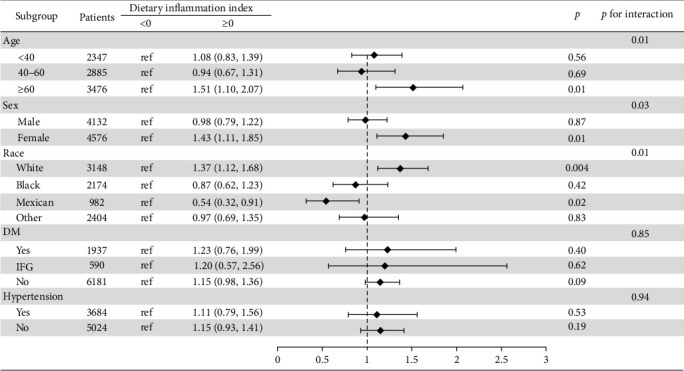
Subgroup analyses of the association between DII and NAFLD.

We found a significant interaction between DII and gender and race (*p*  < 0.05). The positive association between DII and NAFLD appeared stronger in elderly people (aged > 60 years; OR: 1.51; 95% CI: 1.10–2.07); females (OR: 1.43; 95% CI: 1.11–1.85); and whites (OR: 1.37; 95% CI: 1.12–1.68) (Figure 3).

## 4. Discussion

This study delves into the connection between the DII and NAFLD. Our findings reveal a significant link between a proinflammatory diet and an increased risk of NAFLD. Moreover, the most notable correlation between DII and NAFLD emerged among Caucasian women aged over 60 years.

The results of the previous study were similar to the current one, but slightly different. In the study by Tyrovolas et al. [28], four equations were used instead of the diagnosis of NAFLD, and the inflammatory index of diet was not estimated through DII, but through the dietary anti-inflammatory index (D-ALL), which is not a widely recognized scale. Therefore, it seems more prudent to choose DII for estimation purposes. There are also cohort studies by Valibeygi et al. [29] that differ from ours. The NAFLD diagnostic criteria used in the study are currently appropriate. Although liver biopsy is the gold standard for diagnosis, this is not realistic for routine screening of our disease. Vibration-controlled transient elastography (VCTE) is a noninvasive imaging method that can more accurately detect steatosis when it occurs in the liver, and has been widely used in previous studies for the diagnosis of NAFLD [27, 30, 31]. That anti-inflammatory diets are associated with a low incidence of inflammatory diseases seems to be known as common sense, but different parts of the body have different inflammatory mechanisms. Some previous studies have attempted to explain how inflammation in the liver arises. Ahmed et al. suggested that when excessive dietary intake is not consumed in a timely manner, it is converted to fat. Excess free fatty acids and dietary lipids enter the liver and can be deposited as ectopic fat and become lipotoxic, leading to damage to organelles in hepatocytes, and the dysregulated organelles release excessive reactive oxygen species (ROS) and proinflammatory cytokines, leading to systemic inflammation [32]. Excessive intake of proinflammatory diets like high-sugar, high-carbon-water foods, processed meats, etc., are generally converted to fat, and the process described above occurs to drive inflammation in the liver, which in turn leads to NAFLD. Strate et al. [33] found that a proinflammatory diet may lead to chronic low-grade systemic inflammation, which may contribute to the development of NAFLD. This inflammation may be mediated by proinflammatory factors, such as ferritin and CRP [34], and gut microbes may also mediate the link between a proinflammatory diet and NAFLD [35].

Previous research has extensively explored the anti-inflammatory properties of various diets, often utilizing CRP levels as a marker of inflammation. Certain dietary patterns, such as those rich in whole grains [36], fruits, and vegetables [37], have demonstrated the ability to decrease inflammation, as evidenced by lower serum CRP levels. These diets are distinguished by their high content of vitamins, phytochemicals, and fiber. Moreover, consumption of dairy products among animal foods has been associated with a notable reduction in CRP levels [38]. Examining a specific nutrient can offer insights into the impact of individual dietary components on inflammation. However, in real-world dietary contexts, the composition is intricate, with potential interactions between various foods that may act synergistically or antagonistically. Nealed et al. illustrated that adherence to the Mediterranean diet led to a reduction in CRP levels [39]. Similarly, Soltani et al. [40] investigation into the dietary approaches to stop hypertension (DASH) yielded comparable findings. The Mediterranean diet is rich in antioxidants like polyphenols, which play a role in modulating oxidative stress and metabolic inflammation mediated by nicotinamide adenine dinucleotide phosphate oxidase (NADPH) and NF-κB. Additionally, these polyphenols efficiently upregulate the downstream effectors of various proteins, particularly adenosine 5‘-monophosphate (AMP)-activated protein kinase (AMPK). Phosphorylation of AMPK mitigates oxidative stress and inflammation throughout the body, as indicated primarily by decreased production of ROS, proinflammatory cytokines, and adhesion molecule expression [41].

A notable strength of this study lies in the adoption of a contemporary and rational diagnostic tool for NAFLD diagnosis: VCTE. Previous studies relied on ultrasound and liver enzyme tests [42, 43], which are deemed unreliable for detecting liver fibrosis and may introduce misclassification bias. The second strength lies in the utilization of data from NHANES, which employs a robust sampling methodology representative of the population, ensuring broad-reaching conclusions. However, this study has limitations. The dietary data used for calculating the DII were derived from participants' dietary recalls spanning two 24-h periods. While generally consistent, these may deviate from actual dietary habits. Furthermore, despite efforts to control for confounders, unmeasured variables from the NHANES database had to be excluded.

## 5. Conclusion

The study findings revealed an association between DII and the risk of developing NAFLD, with the risk escalating proportionally as DII increased. Dietary management emerges as a crucial strategy in liver disease prevention, particularly for NAFLD.

## Figures and Tables

**Table 1:** Characteristics of the included population.

Variable	Total	NAFLD		*p*-Value
		No	Yes	
Age, mean (S.E)	49.95 (0.48)	45.66 (0.60)	52.96 (0.42)	<0.0001
Sex, *n* (%)	—	—	—	<0.0001
Male	4132 (47.45)	1446 (36.00)	2686 (64.00)	—
Female	4576 (52.55)	1951 (45.90)	2625 (54.10)	—
Race, *n* (%)	—	—	—	<0.0001
White	3148 (36.15)	1181 (40.94)	1967 (59.06)	—
Black	2174 (24.97)	1015 (50.44)	1159 (49.56)	—
Mexican	982 (11.28)	267 (29.15)	715 (70.85)	—
Other	2404 (27.61)	934 (41.35)	1470 (58.65)	—
Marital, *n* (%)	—	—	—	<0.0001
Married	5245 (60.23)	1890 (36.31)	3355 (63.69)	—
Never married	1494 (17.16)	770 (58.38)	724 (41.62)	—
Widowed/divorced/separated	1969 (22.61)	737 (42.30)	1232 (57.70)	—
Poverty, mean (S.E)	3.24 (0.04)	3.28 (0.06)	3.21 (0.05)	0.4
Education, *n* (%)	—	—	—	<0.001
Less than 9th grade	635 (7.29)	196 (33.69)	439 (66.31)	—
9–11th grade	863 (9.91)	337 (39.15)	526 (60.85)	—
High school graduate/GED or equivalent	1996 (22.92)	775 (38.96)	1221 (61.04)	—
Some college	2815 (32.33)	1053 (38.30)	1762 (61.70)	—
College graduate or above	2399 (27.55)	1036 (46.47)	1363 (53.53)	—
BMI, mean (S.E)	29.73 (0.18)	25.83 (0.19)	32.45 (0.21)	<0.0001
DM, *n* (%)	—	—	—	<0.0001
DM	1937 (22.24)	330 (13.13)	1607 (86.87)	—
IFG	590 (6.78)	153 (24.92)	437 (75.08)	—
No	6181 (70.98)	2914 (49.02)	3267 (50.98)	—
Hypertension, *n* (%)	—	—	—	<0.0001
No	5024 (57.69)	2352 (49.82)	2672 (50.18)	—
Yes	3684 (42.31)	1045 (26.28)	2639 (73.72)	—
Smoke, *n* (%)	—	—	—	<0.001
Former	2170 (24.92)	693 (35.00)	1477 (65.00)	—
Never	5372 (61.69)	2156 (42.93)	3216 (57.07)	—
Now	1166 (13.39)	548 (46.48)	618 (53.52)	—
HDL-cholesterol (mmol/L), mean (S.E)	1.38 (0.01)	1.51 (0.01)	1.29 (0.01)	<0.0001
Triglyceride, mean (S.E)	1.60 (0.03)	1.21 (0.02)	1.87 (0.03)	<0.0001

*Note: p*-Value was calculated by weighted linear regression model for continuous variables and weighted chi-square test was performed for categorical variables.

Abbreviations: *n*, number; S.E, standard error.

**Table 2:** Association between DII and NAFLD.

Variable	Number of events	Model 1		Model 2		Model 3	
		95% CI	*p*	95% CI	*p*	95% CI	*p*
Dietary inflammation index							
Proinflammation (DII ≥ 0)	6839	Ref	—	Ref	—	Ref	—
Anti-inflammation (DII < 0)	1869	0.84 (0.73,0.96)	0.02	0.71 (0.62,0.83)	<0.0001	0.85 (0.73,0.99)	0.04
*p* for trend	—	—	0.02	—	<0.0001	—	0.04

*Note:* Model 1: unadjusted model. Model 2: adjustment for age, gender and race. Model 3: adjustment for age, gender, race, marital, education, smoking, diabetes, Hypertension, HDL-cholesterol and triglyceride.

Abbreviations: CI, confidence interval; OR, odd ratio; Ref, reference.

## Data Availability

The laboratory data from our study is publicly accessible online at https://wwwn.cdc.gov/nchs/nhanes/Default.aspx for global data users and researchers.
