# Correction to “Association Between Dietary Inflammatory Index and NAFLD: A Cross‐Sectional Study of the National Health and Nutrition Examination Survey”

**DOI:** 10.1155/mi/9782804

**Published:** 2026-02-16

Y. He, Y. Yang, P. Cheng, W. Zhang, J. Jia, D. Ye, and J. Wang, “Association Between Dietary Inflammatory Index and NAFLD: A Cross‐Sectional Study of the National Health and Nutrition Examination Survey,” *Mediators of Inflammation* 2025, no. 1 (2025): 1–8, https://doi.org/10.1155/mi/4954551.

In the article titled “Association Between Dietary Inflammatory Index and NAFLD: A Cross‐Sectional Study of the National Health and Nutrition Examination Survey,” there were multiple errors. These errors are shown and corrected below:

**Throughout the manuscript:**

Throughout the manuscript, the year range of the data used from the National Health and Nutrition Examination Survey (NHANES) was stated as 2017–2018, which is incorrect.

The correct range is 2017–2020.

**Section 2.1:**

There was an error in the number of patients excluded for missing covariate information (*n* = 459).

The correct number is 3469.

**Figure 1:**

The figure included the incorrect number of patients whose data were used following exclusion of those missing data on cap and DII, as well as patients who were excluded for missing covariate information. The corrected figure is shown below, and is listed as Figure 1:

**Figure 1:**
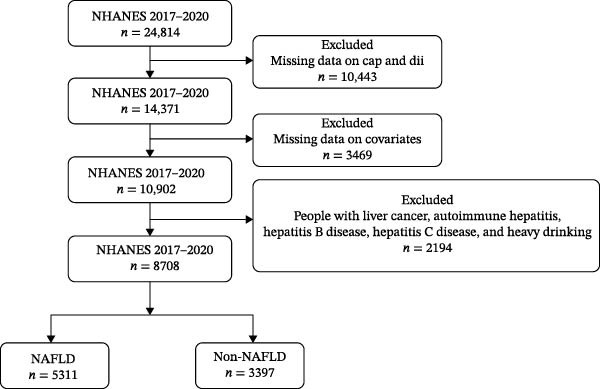
Flow chart of crowd selection. NHANES, National Health and Nutrition Examination Survey. NAFLD, nonalcoholic fatty liver disease.

The authors confirm that the errors do not affect the study’s conclusions.

We apologize for these errors.

